# Reconstruction of injured spinal cord by epigenetic regulation of transplanted neural stem cells

**DOI:** 10.1186/ar3602

**Published:** 2012-02-09

**Authors:** Masahiko Abematsu, Keita Tsujimura, Mariko Yamano, Michiko Saito, Kenji Kohno, Takao Setoguchi, Kazunori Yone, Kinichi Nakashima, Setsuro Komiya

**Affiliations:** 1 Department of Orthopaedic Surgery, Graduate School of Medical and Dental Sciences, Kagoshima University, Kagoshima 890-8520, Japan; 2 The Near-Future Locomotor Organ Medicine Creation Course (Kusunoki Kai), Graduate School of Medical and Dental Sciences, Kagoshima University, Kagoshima 890-8520, Japan; 3 Laboratory of Molecular Neuroscience, Graduate School of Biological Sciences, Nara Institute of Science and Technology, Ikoma 631-0192, Japan; 4 Laboratory of Molecular and Cell Genetics, Graduate School of Biological Sciences, Nara Institute of Science and Technology, Ikoma 631-0192, Japan; 5 Department of Comprehensive Rehabilitation, Osaka Prefecture University, Habikino 583-8555, Japan

## Background

Neural stem cells (NSCs) possess the ability to self-renew and to differentiate into the three major cell types found in the central nervous system (CNS). Recent studies have shown that epigenetic gene regulation events such as DNA methylation and histone modification play important roles in regulating NSC fate specification. In this context, we have previously shown that the histone deacetylase inhibitor valproic acid (VPA) enhances neuronal differentiation of NSCs. Perhaps because these patterns of NSC differentiation are exquisitely controlled during normal embryonic development, restoration of damaged neural networks in the injured adult CNS is severely limited. Here, using a mouse model of spinal cord injury(SCI), we examined the effectiveness of NSC transplantation and differentiation control by VPA administration.

## Materials and methods

NSCs were transplanted into the SCI epicenter 7 days after injury. Non-transplanted control and transplanted mice were then intraperitoneally administered VPA or saline daily, for 7 days, whereafter we monitored their hindlimb motor function using the open field locomotor scale for 6 weeks. We next analyzed the migration, morphology, neuronal marker expression and viability of these cells after co-administration with VPA. We examined extensively the roles of the neurons responsible for reconstruction of broken neuronal networks using two neuronal tracers, immunoelectron microscopy, and two cell-ablation methods.

## Results

We show that transplanting NSCs and administering VPA enhances the functional recovery of their hindlimbs. Neuronal differentiation of transplanted NSCs was promoted in VPA-treated mice. Anterograde corticospinal tract tracing revealed that transplant-derived neurons partially reconstructed the broken neuronal circuits, most likely in a 'relay' manner. Ablation of the transplanted cells abolished the recovery of hindlimb motor function, indicating that transplanted cells contributed directly to the improvement of motor function.

## Conclusions

These data raise the possibility that epigenetic regulation in transplanted neural stem cells can be exploited to provide treatment for SCI.

